# Pregnancy-Specific Stress during the First Lockdown of the COVID-19 Pandemic: Assessing Face-to-Face versus Online Recruitment

**DOI:** 10.3390/ijerph192114102

**Published:** 2022-10-28

**Authors:** Sandra Simó, Juanita Cajiao-Nieto, Natalia V. Awad-Sirhan, Rafael A. Caparros-Gonzalez

**Affiliations:** 1 Department of Psychology, University of Valencia, 46010 Valencia, Spain; 2 Grupo Interdisciplinario de Investigación en Salud, Fundación Universitaria Cafam, Bogotá 111121, Colombia; 3 Facultad de Psicología, Universidad del Desarrollo, Santiago 7610658, Chile; 4 Faculty of Health Sciences, Department of Nursing, University of Granada, 18071 Granada, Spain; 5 Instituto de Investigación Biosanitaria (ibs.GRANADA), 18012 Granada, Spain

**Keywords:** pregnancy-specific stress, COVID-19, online survey, maternal mental health, perinatal mental health, prenatal health

## Abstract

The study aims to assess pregnancy-specific stress among pregnant women in Spain during the first lockdown of the COVID-19 pandemic. Two samples of pregnant women from the south of Spain (Andalusia) were assessed using the Prenatal Distress Questionnaire (PDQ) and a sociodemographic and obstetric questionnaire. Group 1 (N = 155) was recruited face-to-face, whereas Group 2 (N = 78) was recruited online. Pregnancy-specific stress levels were significantly different in both groups. The face-to-face group (Group 1) had higher pregnancy-specific stress levels than the online group (Group 2). The online sample over-represents young adult pregnant women with high education levels and a high number of previous miscarriages. The face-to-face study seems more accessible to racially and ethnically diverse groups. The main concern among both groups was the risk of having a sick neonate. Research during the COVID-19 pandemic can benefit from using online resources to collect data to screen and identify perinatal mental health problems in a crisis environment. Nevertheless, researchers should be aware of the potential limitations this strategy can have, for example, certain groups of people may have limited access to the internet.

## 1. Introduction

Pregnancy is a time of great adaptation for women, which can lead to high stress levels [1,2,3]. The main concerns of pregnant women revolve around their physical status, identity, interpersonal relationships, impending parenthood, childbirth, and newborn’s health [2,3,4]. This particular type of stress in pregnant women is named pregnancy-specific stress. One of the most reliable and valid instruments to measure the pregnancy-specific hassles and women’s perception or response to these is the Prenatal Distress Questionnaire [5,6], used in this study.

Adverse circumstances, whether at times of disaster or not, where a pregnant woman struggles increase the risk of experiencing prenatal stress [7]. On the other side, access to and affordability of health care may help alleviate pregnancy stress [6]. Studies have reported that social support, access to information and a close relationship with midwives and medical staff can reduce the negative effects of prenatal stress [8,9,10]. Age also appeared to be a mediating factor. In this respect, previous studies have reported that a younger maternal age is associated with high maternal psychopathological symptoms [11].

A large body of research establishes that prolonged or intense women perinatal stress can negatively affect maternal health, psychosocial functioning and parenting, and create a permanent imprint on foetal programming and neonatal health [6,7,10,12,13].

Given the potentially harmful consequences of prenatal stress, it is critical to invest effort in screening pregnant women for high stress to efficiently promote their health and well-being and that of their offspring. Moreover, the analysis of prenatal stress and associated risk factors is more dramatic in an exceptional situation such as a pandemic. There is evidence that the pandemic has amplified the general population’s levels of anxiety and depression, and other psychiatric conditions, specifically among pregnant women [14,15,16].

The COVID-19 pandemic caused by the novel SARS-CoV-2 started in 2019 and ignited a global health crisis. Characterized by a progressively emerging life threat [17], this pandemic triggered many changes in economic, socio-cultural, personal and interpersonal relationships. Pregnant women are considered to be one of the most vulnerable groups in this pandemic. The risk of complications associated with SARS-CoV-2 was considered higher in pregnant women [14,18], and the environmental circumstances for pregnant women were extremely adverse and unsupportive. Most of the traditional resources and strategies to monitor pregnancy and prepare women for birth were blocked or delayed in an attempt to constrain the ever-expanding SARS_CoV-2 and to provide spaces to respond to the COVID-19 pandemic. New approaches were introduced, substituting antenatal face-to-face appointments with phone calls, video chats and other online approaches [17,19,20,21].

In light of these considerations, the main objective of this study is to find out the intensity of prenatal stress and the profile of pregnant women during the COVID-19 pandemic. In addition, this analysis will provide further insight into the psychological processes during pregnancy.

In the last decade, pregnant women have increased the use of the internet as a source of health information and social support [19,22,23,24]. This has also encouraged among researchers the use of online resources to recruit pregnant women through web-based participant recruitment platforms or informal social media campaigns [20,25] as a trustful source of data collection.

The use of online social media platforms and services seems to have taken hold during the pandemic [21]. Recent studies evidenced that web-based antenatal care and education reduces pregnancy stress, improves self-efficacy and is an adequate response to the need to support pregnant woman, while curbing the spread of COVID-19 and increasing the sense of safety [12,21]. The circumstances introduced by the COVID-19 outbreak and mobility restrictions have expanded and established the use of the internet and digital media as a trustful source of data collection in healthcare research. Internet-based recruitment and data collection for research have shown to offer benefits in terms of feasibility and accessibility [26,27,28,29].

However, it remains largely unexplored whether data on pregnant women collected through an online survey during this pandemic is equivalent to data collected face-to-face. For this reason, the study’s second goal is to contribute to the discussion about the benefits and limitations of online assessment during the COVID-19 pandemic. To accomplish this goal, we describe the results of two independently designed studies: a group of pregnant women recruited through an online strategy versus a group of pregnant women recruited through a traditional face-to-face strategy. Both studies analysed Spanish women’s prenatal stress with the Prenatal Distress Questionnaire during the pandemic’s first lockdown. This was one of the most critical periods of the pandemic, characterised by severe movement restrictions, a collapsed health system and a remarkable lack of knowledge about the consequences of the infection in both, pregnant women and their foetuses.

In summary, the study has two aims: (a) to evaluate the rate and profile of pregnancy-specific stress during the first lockdown of the pandemic in the South of Spain; and (b) to describe Spanish pregnant women´s prenatal stress levels according to different sample access and data collection strategies.

## 2. Materials and Methods

### 2.1. Individuals

This was a descriptive cross-sectional study with a two-group design. Two research groups contributed to present the study by providing each a sample of pregnant woman from the same living area (Andalusia, South of Spain) during the first lockdown between March and June. Both studies used the same measurement, but different sample recruitment and data collection methods. In the first group, pregnant women were recruited using non-probabilistic sampling of consecutive cases. Pregnant women were invited to participate in a study by a midwife at a face-to-face prenatal care appointment in the public health system (Group 1, N = 155). In the second group, we conducted an internet-based cross-sectional survey. Pregnant women were recruited through social media platforms using a non-probabilistic snowball-type strategy (Group 2, N = 78) and following the criteria established by the Checklist for reporting results of internet E-surveys [30].

Eligible participants in both studies were pregnant women in different gestational stages, aged 18 or older, and living in Andalusia (Southern Spain) who were proficient in reading, writing, and speaking in Spanish language and completed the whole assessment.

All 233 participants filled in the Prenatal Distress Questionnaire (PDQ) and sociodemographic and obstetric information.

### 2.2. Procedure

Face-to-face recruitment procedure (Group 1): Pregnant women were recruited from 15 April to 15 May 2020 during the first COVID-19 lockdown in Southern Spain. Participants were recruited by a midwife at the (blinded for peer review) and a health centre in (blinded for peer review). The midwife offered pregnant women the possibility to participate in this study during an antenatal appointment, presenting the aim of the study, ensuring anonymity and confidentiality of the information, and explaining to potential participants the possibility of leaving the study at any stage. All participants of the study gave their informed consent prior to participating in the study.

Online recruitment procedure (Group 2): Participants were recruited between 23 March and 4 June 2020 for an anonymous and confidential online survey. An advertisement, including an image with a headline, description, website URL and a call to action, was distributed with the help of institutions and perinatal professionals through social networks (Instagram, Facebook, LinkedIn, WhatsApp). The website URL was a link to an online survey created with the LimeSurvey platform that could be completed in approximately 20 min by any pregnant woman living in Andalusia with internet access. All participants of the study gave their informed consent prior to participating in the study.

No incentive was provided to participants for their collaboration.

### 2.3. Instruments

Sociodemographic and obstetric questionnaire: Participants completed an ad hoc questionnaire including sociodemographic information (age, country of origin, marital status, employment status, educational level, direct or indirect contact with COVID-19) and obstetric data (gestational age, type of pregnancy—natural versus assisted reproductive technology, wanted pregnancy, and history of perinatal loss).

The Spanish version of the Prenatal Distress Questionnaire [31] was used to assess the levels of pregnancy-specific stress. The PDQ [5,32] is a scale that provides information on specific worries and concerns women have during pregnancy related to physical symptoms, relationships, parenting, medical problems, birth preparation, and baby’s health. It consists of 12 items structured in three factors: Factor 1 is used to assess worries and concerns related to birth and include item 3 (worry about handling the baby), item 9 (worry about having an unhealthy baby), item 10 (anxious about labour and delivery), item 11 (fear of a potential premature birth) and item 12 (concerns about not becoming emotionally attached to baby); Factor 2 is associated with fears and concerns related to relationships and include item 4 (annoyed about emotional ups and downs), item 5 (troubled that social relationships may change) and item 8 (concerned that relationship with partner will change); Factor 3 is related to worries and concerns associated with the physical state and include item 1 (troubled about weight gain), item 2 (irritated about physical symptoms) and item 7 (bothered about body shape and size). Responses are on a five-point Likert scale ranging from 0 (not at all) to 4 (extremely), with a possible range of 0-48. Early studies using the PDQ inform an M = 14.9 and SD = 7.2 [32]. The Cronbach´s alpha of the full Spanish version of the PDQ was α = 0.743 [31]. Regarding Group 1 (face-to-face), the internal consistency of the PDQ was α = 0.601, while the internal consistency of factors was α = 0.524 (Factor 1), α = 0.452 (Factor 2), α = 0.568 (Factor 3). Regarding Group 2 (on-line), the internal consistency of the PDQ was α = 0.769, while the internal consistency of the factors was α = 0.636 (Factor 1), α = 0.641 (Factor 2), α = 0.607 (Factor 3).

### 2.4. Data Analysis

Data analysis was performed using SPSS software V.26.0 for Mac (SPSS, Armonk, NY). A *p*-value below 0.05 was defined as statistically significant. All percentages for all variables were calculated, and the comparisons between samples were performed using Chi-Square for categorical data and T-Student test [33] for continuous data. Percentage (%) was used to express categorical variables.

Univariate analysis of variance- ANOVA was performed to explore the impact of the type of recruitment (face-to-face versus online) on PDQ scores, followed by a Bonferroni post hoc analysis. Bonferroni analysis is a conservative post hoc procedure designed to compare different combinations while controlling the overall Type I error rate (α) and consequently controlling Type II error rate. Further explanation on Bonferroni post hoc analysis using SPSS software can be found at IBM support [34].

A linear regression analysis was performed to assess whether the age of participants could predict the levels of pregnancy-specific stress (PDQ).

According to the statistical power analysis program G*Power 3.1.3 [35], a preliminary sample size was calculated and considered adequate considering a confidence level of α = 0.05 and a power of 1 − β = 0.8, concluding that the sample size should comprise 68 participants in each group. Group 1 was comprised of 155 participants and Group 2 was comprised of 78 participants.

### 2.5. Ethics

The study was approved by the University of Valencia Ethics Committee (UV- INV_ETICA-1291254) and the Ethics Committee for Human Research in Andalusia (CIV20/00132), and was conducted following the Declaration of Helsinki. All participants voluntarily provided written informed consent to participate in the study.

## 3. Results

### 3.1. Sociodemographic and Obstetric Description

A total of 233 pregnant women participated in this study. Group 1 comprised 155 participants recruited face-to-face. Group 2 included 78 participants recruited using online platforms. The mean age for Group 1 was 32.10 years (SD = 5.67), while in Group 2 the mean age was 33.70 years (SD = 3.99). A between-groups significant difference was found in the following variables: age (*p* < 0.002), country of origin (*p* < 0.01), level of education (*p* < 0.01), and previous miscarriages (*p* < 0.01). Descriptive information regarding Group 1 and Group 2 is shown in Table 1.

### 3.2. Pregnancy-Specific Stress Items between Groups

The total mean score for the PDQ in Group 1 was higher [M = 23.94 (SD = 3.89)] than it was in group 2 [M = 17.10 (SD = 6.74)]. The difference found between groups was significant t = 9.81, *p* < 0.001.

**Figure 1:**
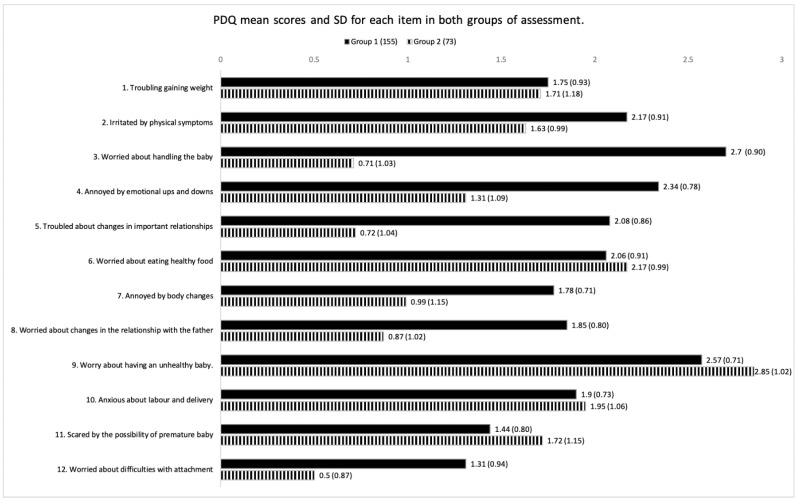
PDQ mean scores and standard deviations (in brackets) for each item in both groups. Group 1 (face to face); Group 2 (on-line).

An exploratory and descriptive analysis identifies the divergence between groups (means and standard deviation) for each question score of the PDQ (see Figure 1). For the face-to-face group (Group 1), the principal concern during the first lockdown of the COVID-19 pandemic was holding the baby when getting home from the hospital. At the same time, participants in Group 2 were mostly concerned about having an unhealthy baby. In both groups, the item assessing worries about difficulties establishing attachment with the newborn obtained a lower score.

### 3.3. Pregnancy-Specific Stress Levels among Group 1 and Group 2 in the First, Second and Third Trimester

The ANOVA analysis revealed an interaction effect between groups in the PDQ scores (F(1, 232) = 96.11, *p* < 0.001, η^2^partial = 0.29). These differences remained significant when including the age of participants in the model (F(1, 232) = 96.11, *p* < 0.001). Bonferroni post hoc analysis on pregnancy-specific stress revealed significant differences in the first (F(1, 27) = 6.10, *p* < 0.05, η^2^partial = 0.19), second (F(1, 77) = 28.91, *p* < 0.001, η^2^partial = 0.27) and third trimester (F(1, 232) = 6.10, *p* < 0.05, η^2^partial = 0.19), and between both groups (F(1, 232) = 67.76, *p* < 0.001, η^2^partial = 0.34).

**Figure 2:**
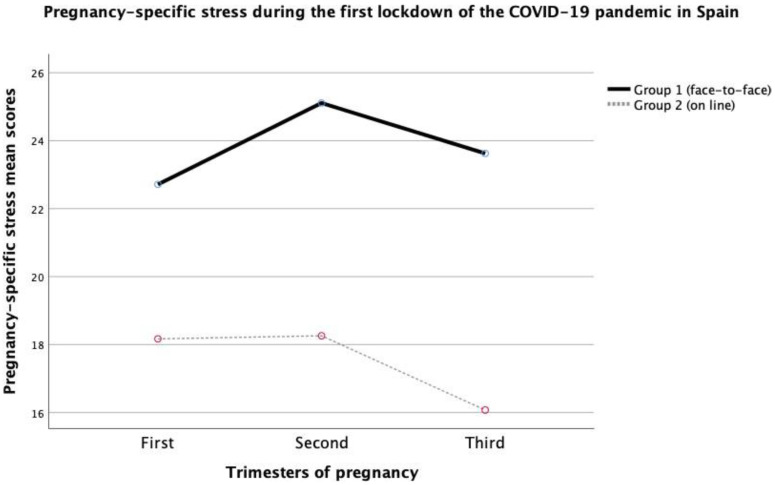
Cross-sectional assessment of pregnancy-specific stress in the first, second, and third trimester (face-to-face versus online recruitment).

Regarding the mean PDQ scores, the face-to-face group (Group 1) had higher PDQ scores in the first (Group 1: M = 22.71, SD = 2.93; Group 2: M = 18.17, SD = 6.67), second (Group 1: M = 25.11, SD = 3.72; Group 2: M = 18.26, SD = 7.37) and third trimester (Group 1: M = 23.63, SD = 4.05; Group 2: M = 16.07, SD = 6.23). The differences between groups were significant in the first (t = 2.47, *p* < 0.05), second (t = 5.37, *p* < 0.001), and third trimester (t = 8.23, *p* < 0.001) (see Figure 2).

A linear regression analysis was performed to assess whether a participant’s age could predict the levels of pregnancy-specific stress. No significant association was found in the full sample of this study or when selecting either Group 1 or Group 2 (*p* < 0.05).

## 4. Discussion

This research was carried out as a result of the cooperation between two research teams that focused primarily on assessing the rate of pregnancy-specific stress using the same measure (PDQ) during the first lockdown of the COVID-19 pandemic in the South of Spain. A severe lockdown characterized this first period, however, all pregnant women continued to be called in for their basic check-ups at their health centre.

On the one hand, the study participants were a group of pregnant mothers recruited directly in a public health centre and hospital during their antenatal appointment; on the other hand, a group of expectant mothers were recruited online via social media and assessed through an internet-based survey. Despite both groups sharing many common characteristics, differences among participants were found in age, level of education, country of origin and history of perinatal loss. In our study, participants in Group 2 (online) were substantially older and had a higher educational level than those in Group 1 (face-to-face). Ouyaba and Kesim [22] obtained a similar profile in a study exploring the effect of the internet on decision-making during pregnancy. In this study, access to the internet and proficiency in computer skills were associated with a higher purchasing power and educational level. A previous study found that younger participants using an online recruitment method were more prone to drop out of the study than older and more educated participants [21,23,36]. According to these studies, older pregnant mothers with a high educational level are more likely to use the Internet to seek information regarding their health and participate in online evaluation and intervention.

Furthermore, as for the participant’s country of origin, the face-to-face group included more migrants than the online counterpart, that is, more pregnant women from diverse racial and ethnical groups coming from countries other than Spain. In our online group, only one woman stated not to be of Spanish origin, contrasting with the general Spanish population, where 11.4% is foreign [37]. In line with our results, other studies also using online surveys on pregnant women in the U.S. found an underrepresentation of women of colour and other racially and ethnically diverse groups [38]. As suggested by this study, migrants can have limited internet access and skills, which may influence the possibility, interest and ability to participate in online recruitment studies [39], but also in virtual online consultations and support.

Another variable found to be different between the two groups was the history of previous miscarriages. Women recruited online reported more previous miscarriages than those in the face-to-face group. We hypothesize that this finding could be associated with the intimacy and sense of security that the online method offers to answer an uncomfortable question anonymously, as pointed out by other authors [40]. In this sense, online recruitment would show an advantage over the face-to-face recruitment of participants by ensuring anonymity. Another possible explanation for this finding could be associated with the fact that an advanced maternal age, which seems to be overrepresented in the online group, is more likely associated with adverse obstetric outcomes such as prematurity, low birth weight or miscarriages [11,41].

In short, our online study over-represents young adult pregnant women with high education levels and a high number of previous miscarriages. The face-to-face study, on the other hand, includes more pregnant women from other countries than Spain.

In respect to the mean PDQ score obtained in this study, the mean score for the PDQ in Group 1 was significantly higher [M = 23.94 (SD = 3.89)] than in Group 2 [M = 17.10 (SD = 6.74)]. The results of Group 2 (online) are similar to the ones reported by Ibrahim and Lobel [6] in their systematic review calculated from 25 studies prior to the pandemic, where they identified mean prenatal stress (PDQ) of 16.21 (SD = 6.22). It also coincides with the results reported by Romero-Gonzalez et al. [42] using an online survey during the pandemic in Spain, where they identified mean prenatal stress (PDQ) of 16.87 (SD= 6.71). Hence, online recruited pregnant women presented similar levels of PDQ to those analysed before the pandemic. Significant higher levels of PDQ were observed in Group 1: pregnant women recruited face-to-face in health centres during Spain´s severest restrictions on individual mobility and highest psychological uncertainty. There is evidence that the pandemic has impacted the general population’ s level of anxiety, specifically among pregnant women, who reported anxiety levels about two-thirds higher than normal [14,15,25,43]. We observe that visiting the health centre at a time of greatest threat and uncertainty may trigger high stress levels and concerns about being exposed to the Coronavirus in the hospital environment, as evidenced in Group 1 results. A study recently developed in Israel found that the use of public transportation, the potential infection of a relative, being in public places, and going for pregnancy check-ups were among the strongest sources of anxiety in pregnant women during the COVID-19 pandemic [44]. Subsequent studies [45,46] focusing on pregnancy-specific stress add new variables to assess this construct and adapt it to the pandemic-related stressors, including concerns regarding pregnancy and childbirth, concerns regarding infections by COVID-19, and even positive appraisals like perceiving benefits of being pregnant during the pandemic.

In relation to the pregnancy-specific stress scores, pregnant women in the face-to-face group cross-sectionally showed high pregnancy-specific stress levels in the first, second, and third trimesters. It was noted that, in both groups, pregnancy-specific stress was high in the first and second trimester and significantly lower in the third trimester. Contrary to expectations, this is an altered pattern since studies with low-risk pregnant women prior to the pandemic indicate that prenatal stress increases during pregnancy and is usually higher in the third trimester [8,42,44]. As these studies show, pregnant women exposed to high levels of stress or those who developed psychopathology during the postpartum period presented higher levels of stress in the third trimester [42,44,47]. Possibly the prenatal stress linked to the experience of pregnancy is minimized by the pressure of the pandemic in the personal, family and socio-economical area.

A more detailed descriptive analysis of the differences found in each item between the two groups reflected that the item receiving the lower score in both groups was item 12 (worries and concerns associated with not being able to bond with the baby). The biggest concern found in the present study in both groups was associated with item 9 (worries and concerns about having an unhealthy baby), probably due to the COVID-19 pandemic and the potential risk of vertical transmission of the SARS-CoV-2 [31]. Our study agrees with a previous study concluding that concerns about the foetus’s health were highly associated with anxiety and psychological distress during the COVID-19 pandemic [44].

Next, we will delve into our second objective of the study, namely, to contribute to the discussion about the benefits and limitations of online assessment in pregnant women during the COVID-19 pandemic.

As our pooled results show, it is important to include measures such as online assessment to improve pregnant women´s psychological well-being in times of crisis. In this sense, we are in line with other studies that highlight the benefits of internet-based recruitment and data collection for research in terms of feasibility and accessibility. We consider digital media a trustworthy source of data collection in healthcare research [26,48]. Online assessment is safer, more practical and economically feasible than face-to-face strategies, especially when describing COVID-19 related attitudes, emotions, and behaviours [49]. Additionally, online assessment can reach underrepresented populations like people in remote geographic locations, from culturally and linguistically diverse backgrounds, or with disabilities [27].

Online approaches to assessing and intervening in pregnant women are undoubtedly opening up a new area of psychological and medical care specialization [12,45,50], which is becoming a feature of future research. First, however, we should point out some conditions when using the online methodology to better understand research results and improve research practices beyond the context of pandemics.

Based on the characteristics of the pregnant mothers recruited online and face-to-face in our studies, we observe that the online study over-represents young adult pregnant women with high education levels and under-represents pregnant women without Spanish origin living in Spain. On the other hand, according to our observations, the face-to-face group of pregnant women includes a greater diversity as regards sociodemographic characteristics, in our case related to age, education, and racial or ethnic origin. The face-to-face study that recruited pregnant women during their appointments in the health centre included more women from diverse racial and ethnic backgrounds. Spanish public healthcare is universal and thereby encompasses a high sociocultural diversity.

These observations suggest that online methodology allows access to a particular group of pregnant women who show interest in participating virtually, with access to virtual surveys and a reliable internet connection. In this regard, multiple studies have documented these technological barriers in studies conducted during the COVID-19 pandemic with adults in developed and developing countries [51,52,53]. Therefore, we conclude that online evaluation and intervention are likely to represent imbalanced cohorts [39]. On the contrary, face-to-face recruitment of pregnant women during the pandemic in Spain appeared to be more representative and included a greater diversity.

At this point, a critical reflection on our experience conducting research with pregnant women during the pandemic with online and face-to-face strategies leads us to raise two considerations. One aspect to consider is that researchers using online recruitment methods for their studies should be mindful of subgroup variability related to sociodemographic and cultural characteristics and tailor their approach considering these variables [51,52,53]. Another point to consider is that more effort, including feasible and meaningful Information and Communication Technologies in the context of healthcare needs to be invested to reduce inequities in terms of access and ability to use physical devices and software, and internet connectivity.

### Limitations

This study has some limitations. At a methodological level, our study is likely impacted by selection bias, privilege, and access to health care. Further randomized trials should compare face-to-face and online assessment in order to delve into the differences between both methods and develop strategies to improve data quality. Studies comparing methods of recruitment (face-to-face versus online) should be performed after the COVID-19 pandemic to assess whether the differences found in this study remain in time.

Furthermore, it should be noted that findings in this study come from a single psychological measure. Future studies should assess whether the recruitment method (face-to-face versus online) has an impact on the levels of stress reported by participants using a wide variety of psychological indicators. Besides, biological measures of stress could be added to rate potential associations between biological measures of stress during pregnancy and participants’ recruitment methods.

## 5. Conclusions

The present study highlights the need to develop appropriate strategies to screen and identify perinatal mental health problems in a critical context. The levels of pregnancy-specific stress during the first lockdown of the COVID-19 pandemic were higher in samples recruited in the clinical setting than those reported using comparable participants before the COVID-19 pandemic [6]. Pregnant women recruited through social media and online surveys presented significantly lower rates of PDQ. The access mode to the sample seems to probably be affecting the reported stress level. In times when in-person contact is advised to be minimal, health care settings may favour online assessments, where possible, to reduce in-person interactions and potentially alleviate associated stress. Future research might examine the impact of online assessment, as compared to in-person, on pregnant women´s stress levels.

The present study supports the utility of internet-based recruitment and data collection as a tool to recruit pregnant women efficiently and effectively for research purposes. Nevertheless, access to the internet can be curtailed for certain pregnant women [28]. This should be considered when collecting and analysing data and interpreting findings from research studies using electronic social media and the internet to recruit pregnant women.

Both recruitment methods, face-to-face and online, can be helpful. During a pandemic, remote data collection provides an opportunity to efficiently and effectively recruit pregnant women for research purposes. Researchers should be aware of the implications of either method when studying stress levels during the COVID-19 pandemic. Furthermore, studies should be clear and transparent when reporting the method of recruitment used.

## Figures and Tables

**Table 1:** Sociodemographic and obstetric characteristics of participants (N = 233).

		Group 1: Face-to-Face Recruitment	Group 2: Online Recruitment	
Sociodemographic Variables		Frequency (%)	Frequency (%)	X^2^
Country of origin	Spain	122 (78.71)	77 (98.72)	^α^ 20.369 **
	Other counties	32 (20.65)	1 (1.28)	
Marital status	Married/Co-habiting	143 (92.26)	74 (94.87)	^α^ 7.763
	Single/Separated/Divorced	12 (7.74)	4 (5.13)	
Employment status	Employed	105 (67.74)	52 (66.67)	0.027
	Unemployed	50 (32.26)	26 (33.33)	
Level of education	Primary school	8 (5.15)	1 (1.28)	^α^ 14.302 **
	Secondary School	78 (50.32)	22 (28.21)	
	University	69 (44.52)	55 (70.51)	
COVID positive	Yes	6 (3.87)	0 (0)	^α^ 4.971
	No	149 (96.13)	78 (100)	
COVID test	Yes	7 (4.52)	0 (0)	^α^ 5.815
	No	148 (95.48)	78 (100)	
Relative infected	Yes	3 (1.94)	5 (6.41)	3.096
	No	152 (98.06)	73 (93.59)	
**Obstetric variables**				
Previous miscarriages	Yes	15 (9.68)	29 (37.18)	25.621 **
	No	140 (90.32)	49 (62.82)	
Wanted pregnancy	Yes	125 (80.65)	61 (78.21)	0.192
	No	30 (19.35)	17 (21.79)	
Type of pregnancy	Natural	142 (91.61)	73 (93.59)	0.284
	Assisted reprod. technology	13 (8.39)	5 (6.41)	
Trimester	First	21 (13.55)	6 (7.69)	3.293
	Second	46 (29.68)	31 (39.74)	
	Third	88 (56.77)	41 (52.56)	
First pregnancy	Yes	82 (52.90)	35 (44.87)	1.339
	No	73 (47.10)	43 (55.13)	

** *p* < 0.01. ^α^ Fisher´s exact test was used.

## Data Availability

Not applicable.
